# Estimating parameters for probabilistic linkage of privacy-preserved datasets

**DOI:** 10.1186/s12874-017-0370-0

**Published:** 2017-07-10

**Authors:** Adrian P. Brown, Sean M. Randall, Anna M. Ferrante, James B. Semmens, James H. Boyd

**Affiliations:** Centre for Population Health Research, Curtin University, Kent Street, Bentley, Western Australia 6102 Australia

**Keywords:** Record linkage, Probabilistic, Privacy, Data quality, Linkage quality

## Abstract

**Background:**

Probabilistic record linkage is a process used to bring together person-based records from within the same dataset (de-duplication) or from disparate datasets using pairwise comparisons and matching probabilities. The linkage strategy and associated match probabilities are often estimated through investigations into data quality and manual inspection. However, as privacy-preserved datasets comprise encrypted data, such methods are not possible. In this paper, we present a method for estimating the probabilities and threshold values for probabilistic privacy-preserved record linkage using Bloom filters.

**Methods:**

Our method was tested through a simulation study using synthetic data, followed by an application using real-world administrative data. Synthetic datasets were generated with error rates from zero to 20% error. Our method was used to estimate parameters (probabilities and thresholds) for de-duplication linkages. Linkage quality was determined by F-measure. Each dataset was privacy-preserved using separate Bloom filters for each field. Match probabilities were estimated using the expectation-maximisation (EM) algorithm on the privacy-preserved data. Threshold cut-off values were determined by an extension to the EM algorithm allowing linkage quality to be estimated for each possible threshold. De-duplication linkages of each privacy-preserved dataset were performed using both estimated and calculated probabilities. Linkage quality using the F-measure at the estimated threshold values was also compared to the highest F-measure. Three large administrative datasets were used to demonstrate the applicability of the probability and threshold estimation technique on real-world data.

**Results:**

Linkage of the synthetic datasets using the estimated probabilities produced an F-measure that was comparable to the F-measure using calculated probabilities, even with up to 20% error. Linkage of the administrative datasets using estimated probabilities produced an F-measure that was higher than the F-measure using calculated probabilities. Further, the threshold estimation yielded results for F-measure that were only slightly below the highest possible for those probabilities.

**Conclusions:**

The method appears highly accurate across a spectrum of datasets with varying degrees of error. As there are few alternatives for parameter estimation, the approach is a major step towards providing a complete operational approach for probabilistic linkage of privacy-preserved datasets.

## Background

Record linkage is a process that allows us to gather together person-based records that belong to the same individual. In situations where unique identifiers are not available, personally identifying information such as name, date of birth and address are used to link records from one or more data collections. As administrative collections typically capture information for large portions of the population, the linked data allows researchers to answer numerous health questions for the whole population at relatively low cost.

### Privacy-preserving record linkage

Legal, administrative and technical issues can prevent the release of name-identified data for record linkage. New methods have emerged that do not require the release of personally identifying information by data custodians; rather, data custodians use specific encoding processes to transform personally identifying information into a permanently non-identifiable state (an irreversible ‘privacy-preserved’ state). These methods are collectively referred to as privacy-preserving record linkage (PPRL). Under a trusted third party linkage model [1], this operation occurs *before* the release of any data to record linkage units. Thus, personally identifying information is not disclosed by the data custodian. These PPRL methods can be used within existing record linkage frameworks, and are subject to some of the same challenges [2].

One of the most promising PPRL techniques to emerge is a method which uses Bloom filters in record linkage [3]. A Bloom filter is a probabilistic data structure originally developed to check set membership that can also be used to approximate the similarity of two sets. The ability to provide similarity comparisons on two sets of data is highly desirable for accurate record linkage.

An evaluation of Bloom filters in large-scale probabilistic record linkage has shown high linkage quality (equal to that achieved with unencrypted linkage) with relatively good efficiency [4]. This evaluation utilised single field Bloom filters as opposed to record-level Bloom filters, where all identifiers are added into a single Bloom filter [5]. One of the outstanding challenges for a practical probabilistic PPRL approach is to accurately estimate parameter settings [4]. Typical methods to estimate parameters involve manually examining small samples of data. In the privacy-preserving case, this data is not available to examine so alternate parameter estimation methods are required.

### Probabilistic record linkage

**Fig. 1:**
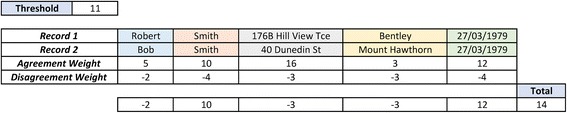
Record comparison example

In probabilistic record linkage, individual records are compared on a pairwise basis. This process makes the number of possible comparisons extremely large for all but small data files. To reduce computation overhead, records are usually only compared if they have information in common i.e. they have the same value in a particular field or set of fields. Known as blocking, this method reduces the computational comparison space. Pairs of records in each block are compared and assessed through comparison of the values in each matching field (e.g. first name, surname, address, etc.). As shown in Fig. 1, each field comparison is assigned a field score, the value of which depends on whether the field value agrees or disagrees. These agreement and disagreement scores (weights) are computed separately for each field. All field scores are then summed to produce a final score. If this score is greater than a set threshold value, the record pair is designated a match. The set of fields used in the linkage are chosen based on characteristics such as completeness, consistency and discriminating power within each dataset. The discriminating power is a measure of entropy, indicating how useful an identifier might be in the record linkage process [6, 7].

In the Fellegi-Sunter model of record linkage [8], the agreement and disagreement scores used in field comparisons are based on the calculation of two specific probabilities, called the m-probability and u-probability [8]. The m-probability is the likelihood of two fields matching if the records belong to the same individual. The u-probability is the likelihood of two fields matching if the records do **not** belong to the same individual. These two probabilities are converted into agreement and disagreement weights for each field as follows:

$$ Agreement\  Weight= \log \left(\frac{m}{u}\right), Disagreement\  Weight= \log \left(\frac{1- m}{1- u}\right) $$

The Fellegi-Sunter model incorporates a simplifying assumption where the chances of agreement or disagreement for one field is independent of the chances of agreement or disagreement for another field [8]. This independence assumption allows us to calculate agreement and disagreement weights for each field separately. Extensions to the Fellegi-Sunter model have been developed for approximate comparisons, allowing the assignment of a partial weight for partial agreement that lies somewhere between agreement and disagreement [9]. While there are many types of approximate comparisons for various types of data, most deal with the distance between two strings [10–12]. To fit these approximate comparisons into a probabilistic model, the distance is converted into a partial weight [13].

Missing values can be problematic in probabilistic record linkage. Comparisons are typically treated in one of three ways: a missing value is assigned the disagreement weight, a zero weight, or a separate weight accounted for explicitly. The last option extends the independence assumption to include probabilities for missing values, altering the calculations for weights. Other approaches involve removing the field from matching or even removing the entire record [10, 14].

### Parameter estimation

Several methods have been developed to estimate m- and u-probabilities [15, 16]; in practice, most methods are based on investigations around data quality and prior knowledge, such as the iterative refinement procedure [17].

Automated methods for deriving m-probabilities, such as through EM (expectation-maximisation) estimation have been devised [16, 18, 19]. The EM algorithm has the potential to provide accurate estimates for m-probabilities, in some cases outperforming the probabilities obtained via the iterative refinement procedure [13]. Other estimation methods do exist, such as an algebraic solution by Fellegi and Sunter [8] and the IMSL routine ZXSSQ (an implementation of the Levenberg-Marquardt algorithm) [20]; however, these are more sensitive to initial parameters and require adjustment functions to keep estimates within bounds [21]. An extensive analysis of parameter estimation techniques for the Fellegi-Sunter model of linkage has been detailed by Herzog et al. [15].

Determination of the appropriate threshold setting above which to accept record-pairs as valid matches typically occur through manual inspection of record-pairs within a range of weight scores [22]. The use of PPRL methods within a probabilistic linkage framework, where only encrypted identifiers are used for linkage, preclude the use of any manual, clerical review and so must rely on the use of alternative, computerised methods to determine the best cut-off values. This ability to correctly estimate parameters is of paramount importance if PPRL techniques are to be practical [4].

In this paper, we present a method for accurately estimating probabilities and an optimal threshold cut-off value that can be applied when using Bloom filters within the Fellegi-Sunter model for record linkage. The work builds on a previous privacy-preserving study, which utilised a probabilistic record linkage framework [4]. In this paper, we evaluate our parameter estimation method in two ways: firstly, in a simulation study using synthetic datasets with varying degrees of error; and secondly, on three large-scale administrative datasets, comparing the resultant linkage quality against the quality achieved using calculated m- and u-probabilities.

## Methods

### Simulation study using synthetic datasets

A series of synthetic datasets were created for our simulation study. Firstly a single ‘master’ dataset was created, containing 1 million records, with multiple records belonging to the same individual. This dataset did not contain any missing values, or errors typical of what would be seen in administrative data. Then, a series of new datasets were created by first taking the error-free master dataset, and removing or degrading the quality of particular fields.

The synthetic data was generated using an amended version of the FEBRL data generator [23]. The distribution of duplicate records (how many records pertain to each individual) was based on the distribution found in the Western Australian hospital morbidity data collection. The values found in the master dataset were based on frequency distributions found in the Western Australian population. Each record in the dataset contained first name, middle name, surname, sex, date of birth, address, suburb, and postcode information. Address information was randomly selected from the National Address File, a public dataset containing all valid Western Australian addresses.^1^

Additional ‘corrupted’ datasets were created by modifying the master dataset with a set level of error. In the 1% error file, 1% of field values to be used for linkage were randomly selected to have their values set to missing; a further 1% were randomly selected to have their values corrupted, through the use of typographical errors, misspellings, truncation and replacement of values. In this way, each record could potentially have multiple fields set to missing or corrupted. The same procedure was used to generate a 5% error file, 10% error file and 20% error file. A privacy-preserved version of each dataset was created, using single field Bloom filters.

### Testing using administrative datasets

Three datasets comprising real administrative data (hospital admissions records from New South Wales (NSW), Western Australia (WA) and South Australia (SA)) were used to demonstrate the applicability of the method to real-world data. These datasets have previously been de-duplicated to a very high standard using full identifiers. The results of those de-duplication linkages are used in this study and act as our ‘truth set’. The information in this ‘truth set’ was not used during the linkage process or the estimation of parameters, but was used only as a standard by which to evaluate our results. This data was made available as part of the Population Health Research Network Proof of Concept 1 project [24].

Privacy-preserved versions of each administrative dataset were created, using single field Bloom filters, in the same way as the synthetic datasets. Due to the size of these administrative datasets, five samples (a random 10%) of each privacy-preserved dataset were created; probabilities are estimated for each sample. A de-duplication linkage was performed on each sample and also against the full dataset. The resulting quality was calculated using the ‘truth set’.

### Application of Bloom filters

The privacy-preserved versions of the synthetic and administrative datasets were created using Bloom filters. Bloom filters were constructed in line with previous work [3]. An empty (or missing) field in the original datasets was left as empty in the privacy-preserved versions.

Matching strategies used for the datasets were based on the strategies used in a published evaluation of linkage software [25]. Two blocking strategies were used; last name Soundex with first name initial, and date of birth with sex. The matching identifiers included Bloom filters for names, address and suburb, using the Sørensen-Dice coefficient comparison for similarity [3]. Sørensen-Dice coefficient values are converted to partial agreement values using a piecewise linear curve, created using Winkler’s [13] method. All other fields, including blocking variables, which are created at the same time as the Bloom filters, used exact matches on cryptographically hashed values. Missing value comparisons were assigned a zero weight.

### Measuring linkage quality

In line with earlier work [3, 26], we used precision, recall and F-measure as our linkage quality metrics. Precision (also known as positive predictive value) measures the proportion of true positive pairs (correct matches) found from all *classified* matches. Recall (also known as sensitivity) measures the proportion of true positive pairs found from all *true* matches. Both precision and recall return a score between 0 and 1, with higher scores indicating less false positives and false negatives (missed matches) respectively. The F-measure is the harmonic mean between precision and recall, providing a single figure with which we can compare results. Typically, a middle-ground is sought between precision and recall, as there is a trade-off between these values. As the probabilistic linkage threshold is increased, the number of false positives decreases (and so precision increases); however, the number of correct matches missed will also increase, leading to a decrease in recall.

The calculations for these metrics are provided below.

$$ Precision=\frac{True\  Positives}{True\  Positives+ False\  Positives} $$

$$ Recall=\frac{True\  Positives}{True\  Positives+ False\  Negatives} $$

$$ Fmeasure=\frac{2\times Precision\times Recall}{Precision+ Recall} $$

### Estimating m and u probabilities

The EM algorithm has been used to calculate the m-probabilities (***m*** *)*, u-probabilities (***u***) and the proportion (***p***) of record pairs that match in probabilistic linkage [21]. It is an iterative algorithm that uses the output values of one iteration as the input to the next. We added two additional variables to the EM algorithm as described by Jaro [21], the *missing m-probability* and *missing u-probability* values (denoted by ***m***_***m***_ and ***u***_***m***_ respectively), to more accurately estimate a single threshold cut-off value (discussed later).

Jaro [21] suggests the algorithm is not particularly sensitive to the starting values (***m***, ***u***, ***m***_***m***_ *,* ***u***_***m,***_ ***p***). However, the starting values for ***m*** should be higher than those for ***u***. We thus set an initial value of 0.1 for ***m***_***m***_ and ***u***_***m,***_ 0.8 for ***m*** and 0.1 for ***u***.

Given two files, A and B, we began by iterating through all possible combinations of field comparisons between A and B. The count of each field state combination was tabulated (an example is shown in Table 1). There are, at most, 3^n^ possible field state combinations for *n* fields, assuming each field either agrees, disagrees or is missing. The ‘missing’ state occurs when a pairwise comparison involves a missing or empty value.

**Table 1:** Field state combinations

First Name	Last Name	Sex	Year of Birth	Count
Agree	Agree	Agree	Agree	1502
Agree	Agree	Missing	Disagree	2142
Agree	Disagree	Disagree	Missing	28,644
…	…	…	…	…

The first part of the EM algorithm is the expectation step. For each field state combination, we calculate *recall* and *false positive rate (fpr)*. For *recall*, each agreement in the table is replaced with ***m***, each disagreement with (1 – ***m***_***m***_ – ***m***), and each missing with ***m***_***m***_. The product of these is the *recall* for that field state combination. Similarly, for the *fpr*, each agreement in the table is replaced with ***u***, each disagreement with (1 – ***u***_***m***_ – ***u***) and each missing with ***u***_***m***_ *.* The product of these provides the *fpr*.

The *recall* and *fpr* allow us to calculate the proportion of true matches for each field state combination *j*:

$$ {p}_j=\frac{{p\bullet recall}_j}{\left({p\bullet recall}_j\right)+\left(\left(1- p\right)\bullet {fpr}_j\right)} $$

The maximisation step involves the calculation of ***m***, ***u***, ***m***_***m***_, ***u***_***m***_ and ***p***. The ***m*** value for each field is calculated as the ratio of true matches that ‘agree’ for that field to the total true matches. Likewise, the ***u*** value for each field is calculated as the ratio of false matches that ‘agree’ for that field to the total false matches. The ***m***_***m***_ and ***u***_***m***_ values use the ratio of matches that are ‘missing’.

The output values of (***m***, ***u***, ***m***_***m***_, ***u***_***m,***_ ***p***) are then used as the input into the next iteration. Iterations are run until values converge. Convergence will occur when the output values differ only minimally from the input values.

### Determining a threshold/cut-off setting

In addition to estimating probabilities for a probabilistic linkage, it is important to specify a threshold value that provides optimal resultant linkage quality.

Using the information generated during the EM step, we can estimate the quality of linkage for every combination of weights between a range of possible threshold values (i.e. using precision, recall and F-measure). However, the table of field state combinations used for the EM step only contains field state combinations that were present in the datasets A and B. The *full* set of possible combinations is required to calculate a suitable threshold setting. Field state combinations that are not present in the field state combination table were added with a count of zero, and *recall* and *fpr* were calculated.

Using the full field state combination set, we calculated the weight for each field state combination. Each agreement entry in the table was replaced with the corresponding agreement weight for that field using ***m*** and ***u*** calculated by the EM algorithm. Likewise, each disagreement entry was replaced with the disagreement weight for that field using the same ***m*** and ***u*** *.* Each ‘missing’ entry was replaced with a weight of zero.

To estimate precision, recall and F-measure, we calculated the *True Positives* and *False Positives* for every field state combination. For these estimations, we required the total *True Matches* (true positives and false negatives) and *False Matches* (true negatives and false positives). The total *True Matches* was estimated as part of the EM algorithm, and thus we used the value calculated in the final iteration of the maximisation step. The total *False Matches* was re-estimated as the *total comparison space* less the *True Matches.*

For a single file de-duplication, the total comparison space is:

$$ \mathrm{total}\ \mathrm{comparisons}=\left(\frac{\mathrm{RecordCount}\times \left(\mathrm{RecordCount}-1\right)}{2}\right) $$

To calculate the *True Positives* and *False Positives*, we multiplied the *recall* and *false positive rate* for each field state combination by the total *True Matches* and *False Matches* respectively.

$$ {True\  Positives}_j= True\  Matches\bullet {recall}_j $$

$$ {False\  Positives}_j= False\  Matches\bullet {fpr}_j $$

We calculated the *True Positives* and *False Positives* for each field state combination so that *precision* could be estimated. To calculate the *precision* for a particular threshold, each field state combination with a weight above that threshold value had their *True Positives* and *False Positives* summed before *precision* was estimated.

We did not calculate *False Negatives,* as this can be derived from the total *True Matches* (*True Positives* plus *False Negatives*) value calculated earlier to estimate *recall*. To calculate *recall* for a particular threshold, the *True Positives* were summed from values for each field state combination that have a weight above that threshold.

As the computation requirements for calculating precision, recall and F-measure are relatively low; we calculated these for all possible weight combinations. With a list of threshold values and corresponding *precision*, *recall* and *F-measure* values, we were able to determine an optimal threshold value for each linkage (i.e. the single threshold score with the highest estimated *F-measure*).

### Evaluation of parameter and threshold estimation

For each version of the synthetic datasets, and additionally, for the administrative datasets, probabilities for ***m*** and ***u*** were estimated together with a threshold cut-off value. The EM algorithm was used to estimate ***m*** only for each de-duplication linkage. The frequencies used for our EM algorithm were calculated on blocks, and as such, the number of non-matches observed was greatly reduced, thereby introducing an undesirable bias into the EM algorithm’s ***u*** estimates [21]. Consequently, we elected to use Jaro’s u-probability estimate (on unblocked data) ***u***, together with the EM algorithm’s estimated ***m*** value.

As part of our simulation study, a de-duplication linkage was run on each synthetic dataset using this combination of values, and a linkage was also run using calculated m- and u- probabilities. Optimal threshold values were estimated for both sets of probabilities. The highest F-measure and estimated threshold F-measure were recorded and compared for all synthetic dataset de-duplication linkages. Similarly, in our test using real data, de-duplication linkages were run on the administrative data; calculated m- and u- probabilities were obtained using the administrative data ‘truth sets’. The accuracy of the probability estimates on the administrative dataset samples was measured using the root-mean-square error (RMSE), comparing the F-measure obtained from the EM algorithm probabilities with that obtained from calculated probabilities. RMSE was also used to compare the F-measure obtained at the estimated threshold with that which would be obtained at the correctly chosen threshold. The formula used was as follows:

$$ RMSE=\sqrt{\frac{1}{n}\sum_{Dataset\ 1}^{Dataset\  n}{\left({Fmeasure}_{estimated}-{Fmeasure}_{actual}\right)}^2} $$

## Results

### Synthetic data

The characteristics of the synthetic datasets are shown in Table 2. As the dataset error rates increase, the number of unique values for each field increases significantly because of the corruption introduced during dataset creation. The discriminating power for each field also increases with the simulated data corruption.

**Table 2:** Synthetic dataset characteristics

Field	0% Error		1% Error		5% Error		10% Error		20% Error	
	Unique Values	Discriminating Power	Unique Values	Discriminating Power	Unique Values	Discriminating Power	Unique Values	Discriminating Power	Unique Values	Discriminating Power
First Name	31,183	8.91	34,595	8.92	45,914	8.99	58,046	9.08	78,256	9.29
Middle Name	25,002	7.33	28,224	7.35	38,285	7.45	48,973	7.59	67,160	7.95
Last Name	56,507	10.87	61,198	10.88	77,088	10.96	94,925	11.07	125,483	11.35
Dob Year	112	6.49	114	6.49	116	6.50	117	6.51	119	6.53
Dob Month	12	3.58	12	3.58	12	3.58	12	3.58	12	3.58
Dob Day	31	4.94	31	4.94	31	4.94	31	4.94	31	4.93
Sex	2	1.00	2	1.00	2	1.00	2	1.00	2	1.00
Address	171,088	12.89	178,583	12.92	207,909	13.04	241,966	13.21	304,353	13.66
Suburb	1962	8.33	7390	8.36	19,664	8.48	31,054	8.65	49,929	9.10
Postcode	379	6.77	1755	6.80	2579	6.91	2981	7.06	3395	7.45

The results from de-duplication linkages of the synthetic datasets using calculated probabilities and EM probabilities are shown in Table 3. These results show that the use of EM for probability estimation, combined with our threshold estimation technique, provided linkage quality comparable to the best achievable using calculated probabilities, on data with up to 20% error.

**Table 3:** Synthetic dataset linkage quality - estimated vs. calculated

Data Error Rate	Calculated Probabilities				EM m-probs and Estimated u-probs			
	Highest		Estimated		Highest		Estimated	
	Threshold	FMeasure	Threshold	FMeasure	Threshold	FMeasure	Threshold	FMeasure
0%	49	1.0000	8	0.9999	49	1.0000	8	0.9999
1%	9	0.9979	16	0.9978	13	0.9979	11	0.9979
5%	8	0.9549	16	0.9541	12	0.9549	11	0.9549
10%	8	0.8443	16	0.8399	12	0.8439	11	0.8436
20%	8	0.5217	16	0.4938	12	0.4999	11	0.4917

As one would expect, de-duplication of the master dataset (without error) produced a perfect result with F-measure of 1.0 at a threshold of 49 (the sum of all agreement weights for each field). The use of EM estimated m-probabilities produced the same result. However, estimation of a threshold value for the master dataset was significantly lower, with a value of 8 for both calculated and estimated probabilities. Note, however, that although this threshold estimate is much lower, it results in just 60 false positives from the entire comparison space, giving an F-measure of 0.9999995.

While it is possible for the threshold to be estimated to one or two decimal places, the use of a whole number here was made for simplicity. It is possible that a better estimate could be made with a finer precision but the differences between thresholds shown here using whole numbers are already negligible.

As Table 3 shows, using our estimation technique, there is a slight decrease in linkage quality as error rates in the data increase (i.e. for 1% error, an F-measure of 0.9979 vs. 0.9979, compared to 20% error with an F-measure of 0.5217 vs. 0.4917). However, even at 10% error, the difference is very small with an F-measure of 0.8443 vs. 0.8436.

### Administrative data

The characteristics of the fields in each administrative dataset, such as the number of unique values, missing percentage, and discriminating power were recorded, shown in Table 4. The random samples generated for each administrative dataset were highly representative of the full dataset.

**Table 4:** Administrative dataset characteristics

	NSW(13,534,177 records)			SA(2,509,914 records)			WA(6,772,949 records)		
Field	Unique Values	Missing %	Discriminating Power	Unique Values	Missing %	Discriminating Power	Unique Values	Missing %	Discriminating Power
First Name	168,766	2.9%	8.61	124,849	5.5%	9.18	78,992	0.3%	8.54
Middle Name	114,686	54.2%	6.96	22,180	75.4%	7.19	61,241	40.8%	7.13
Last Name	291,595	0%	10.92	81,431	5.3%	10.81	123,481	0%	10.73
Dob Year	123	0%	6.47	115	0%	6.45	118	0%	6.39
Dob Month	12	0%	3.58	12	0%	3.58	12	0%	3.58
Dob Day	31	0%	4.94	31	0%	4.94	31	0%	4.94
Sex	2	0%	1.00	2	0%	1.00	2	0%	0.99
Address	3,084,889	1.5%	16.96	690,615	8.1%	14.92	1,350,796	0.2%	16.05
Suburb	49,843	0.5%	9.30	10,729	6.9%	7.85	5542	0.1%	7.73
Postcode	3947	0.8%	8.17	2238	8.5%	6.90	2319	0.2%	6.58

### Linkage quality from EM estimates

The estimated m- and u-probabilities of the samples reflect the characteristics described above, with negligible differences observed between the samples for each dataset. The estimated probabilities for each dataset are shown in Table 5.

**Table 5:** Estimated probabilities

	NSW		SA		WA	
Field	EM m-prob	Est. u-prob	EM m-prob	Est. u-prob	EM m-prob	Est. u-prob
First Name	0.9817	0.0024	0.8707	0.0015	0.9732	0.0027
Middle Name	0.4686	0.0017	0.1846	0.0004	0.4385	0.0025
Last Name	0.9916	0.0005	0.8931	0.0005	0.9823	0.0006
Dob Year	0.9973	0.0113	0.9997	0.0114	0.9935	0.0119
Dob Month	0.9987	0.0834	0.9988	0.0834	0.9949	0.0835
Dob Day	0.9965	0.0325	0.9988	0.0325	0.9963	0.0326
Sex	0.9999	0.5008	1.0000	0.5010	0.9998	0.5018
Address	0.8325	7.99E-06	0.6486	2.8E-05	0.7338	1.7E-05
Suburb	0.9303	0.0016	0.7462	0.0038	0.8402	0.0047
Postcode	0.9540	0.0034	0.7574	0.0070	0.8640	0.0104

Comparisons of linkages using the calculated probabilities and the EM m-probabilities with estimated u-probabilities are shown in Table 6. The highest F-measure obtained using the estimated probabilities was slightly higher than that achieved using calculated probabilities in all cases.

**Table 6:** Linkage quality (max F-measure) – EM vs. calculated

Dataset	Probabilities	Sample 1	Sample 2	Sample 3	Sample 4	Sample 5	RMSE
NSW	Calculated	0.9941	0.9943	0.9942	0.9941	0.9940	
	EM	0.9961	0.9965	0.9963	0.9963	0.9961	0.0021
SA	Calculated	0.9532	0.9521	0.9529	0.9553	0.9532	
	EM	0.9590	0.9567	0.9563	0.9582	0.9589	0.0046
WA	Calculated	0.9907	0.9904	0.9910	0.9905	0.9906	
	EM	0.9920	0.9916	0.9921	0.9917	0.9918	0.0012

### Accuracy of threshold estimation

The quality of linkage using the F-measure at the estimated threshold is compared to the highest F-measure for each sample, as shown in Table 7. The RMSE values for each dataset were 0.0019 for NSW, 0.0001 for SA and 0.0046 for WA. The estimated threshold value was slightly below the best threshold for each dataset.

**Table 7:** Linkage quality – max F-measure vs. F-measure at threshold estimate

Dataset	Threshold		Sample 1	Sample 2	Sample 3	Sample 4	Sample 5	RMSE
NSW	Best	14	0.9961	0.9965	0.9963	0.9963	0.9961	
	Estimated	12	0.9943	0.9946	0.9945	0.9944	0.9942	0.0019
SA	Best	13	0.9590	0.9567	0.9563	0.9582	0.9589	
	Estimated	12	0.9589	0.9566	0.9563	0.9581	0.9588	0.0001
WA	Best	13	0.9920	0.9916	0.9921	0.9917	0.9918	
	Estimated	11	0.9871	0.9870	0.9873	0.9871	0.9875	0.0046

## Discussion

In our simulation study, the use of the EM algorithm to estimate probabilities for a de-duplication linkage produced results comparable to those produced by calculated probabilities, even with synthetic datasets that contained 20% introduced error. Similarly, in our tests using administrative datasets, the probability and threshold estimation technique produced very high-quality linkage results. In comparison to the quality of linkage using calculated probabilities, the probabilities used from the EM algorithm produced linkage quality of the simulation datasets that was comparable to the best possible. However, we found better quality results using estimated probabilities on the real administrative datasets, at least in regards to F-measure. This is a somewhat surprising result, and why this occurred for all three administrative datasets is not entirely clear. A recent analysis of the popular F-measure metric suggests that it may not provide a fair comparison between linkage methods if the selected thresholds produce a different number of predicted matches [27]. This behaviour is one possible explanation for our results, and future work will consider additional metrics for measuring linkage quality. It should be noted that the differences between the linkage quality results were relatively small, and we would not expect this to be the case for datasets of all sizes and quality.

The original unencrypted versions of these datasets had previously been linked by Boyd et al. using probabilities estimated with knowledge of previous linkages and refinement through pilot linkages [24]. The probabilities derived from the EM algorithm produced a higher F-measure for both the NSW (0.996 vs. 0.995) and WA (0.992 vs. 0.990) Bloom filter datasets; data for the unencrypted SA dataset was unavailable. On face value, at least, these results indicate that use of the EM algorithm for probability estimation is a viable option, especially where sampling techniques for estimation are not available due to the privacy-preserved nature of the data.

Our study found that the m-probabilities estimated via the EM algorithm did not necessarily match the calculated m-probabilities for each field; however, there was a general consistency of the m-probabilities across all fields. Both our synthetic datasets and the administrative datasets contained many matches and were thus good candidates for probabilities estimated through the EM algorithm. The EM algorithm is known to perform poorly with datasets that have too few matches [15]. Being able to identify and address this issue for privacy-preserved data will require further research.

Our threshold estimation technique also returned very good linkage quality, with a resulting F-measure that consistently approached the best F-measure achievable given the probabilities used. To our knowledge, no alternative method of estimating thresholds exists for use with privacy-preserved data. Without the ability to provide any manual review post-linkage, it is important to be able to estimate a single accurate threshold cut-off value. As such, this technique should be considered for use with Bloom filters for probabilistic linkage.

The threshold values estimated in our study were consistently higher than the optimum threshold when using the calculated probabilities, with fewer false positives and more false negatives returned in each of the linkages (with the exception of the ‘perfect’ synthetic dataset). Interestingly, we found the opposite to be true when using the estimated probabilities, with a consistently lower threshold. Additional simulation studies may help to understand this effect and improve the estimation accuracy. This effect may be a result of the blocking technique used to gather field state combinations and the similarities in the estimation methods for both probabilities and threshold. Although it may be possible to adjust for this underestimation, an advantage of using a lower threshold is that alternative approaches can be implemented which specifically target false positive matches. It may be possible to run automated clerical review procedures on the results, such as graph theory techniques, to find and correct false positive errors [28]. The effectiveness of these techniques on privacy-preserved data is unknown, however.

Future research will examine the use of the EM algorithm on composite Bloom filters. While single field Bloom filters provide excellent quality with probabilistic linkage, they may not provide a sufficient level of privacy for some stakeholders. As such, the use of composite Bloom filters may be necessary. Row-level Bloom filters would not be viable; at least two fields are required for probabilistic record linkage. However, multiple Bloom filters comprising two or three fields may function sufficiently. The use of the EM algorithm and the threshold estimation technique on Bloom filters comprising two or more fields is untested, and more research into the performance of the EM algorithm on data containing composite fields is warranted.

Finally, it is worth noting that the EM algorithm and threshold estimation technique described in this paper have wider application and could be used for any probabilistic linkage (encrypted and unencrypted), not just Bloom filters for PPRL. Provided the datasets being linked have sufficient matches, the estimation technique will produce optimal m-probabilities and a suitable threshold cut-off for the linkage. The u-probabilities can be estimated using Jaro’s estimation method. Unencrypted linkages would benefit from this technique as well, providing a strong empirical foundation from which to build a robust linkage strategy.

## Conclusions

Previous evaluations have shown that privacy-preserving record linkage can be as accurate as traditional un-encoded linkage. An important element in developing a practical probabilistic privacy-preserving approach is to determine how to appropriately set parameters without recourse to manual inspection or prior knowledge of data. As we have shown, use of the EM algorithm and our threshold estimation technique provides a robust method of estimating parameters for probabilistic linkage of Bloom filter datasets. This method appears highly accurate on datasets with varying error levels. Further testing is required on real-world datasets with poorer quality data and on datasets with fewer potential matches. The ability for these techniques to produce consistently accurate results on a variety of data will determine whether they are viable in an operational setting.

## Abbreviations

EM: Expectation-maximisation
FPR: False positive rate
NSW: New South Wales
PPRL: Privacy-preserving record linkage
RMSE: Root mean square error
SA: South Australia
WA: Western Australia

## Footnotes

1 Available from https://data.gov.au/dataset/geocoded-national-address-file-g-naf
